# Advances in neuroprosthetic management of foot drop: a review

**DOI:** 10.1186/s12984-020-00668-4

**Published:** 2020-03-25

**Authors:** Javier Gil-Castillo, Fady Alnajjar, Aikaterini Koutsou, Diego Torricelli, Juan C. Moreno

**Affiliations:** 1 Neural Rehabilitation Group, Cajal Institute, Spanish National Research Council (CSIC), Av. Doctor Arce, 37, 28002 Madrid, Spain; 2 College of Information Technology (CIT), The United Arab Emirates University, P.O. Box 15551, Al Ain, UAE

**Keywords:** Functional electrical stimulation, Neuroprosthetics, Foot drop syndrome, Gait

## Abstract

This paper reviews the technological advances and clinical results obtained in the neuroprosthetic management of foot drop. Functional electrical stimulation has been widely applied owing to its corrective abilities in patients suffering from a stroke, multiple sclerosis, or spinal cord injury among other pathologies. This review aims at identifying the progress made in this area over the last two decades, addressing two main questions: What is the status of neuroprosthetic technology in terms of architecture, sensorization, and control algorithms?. What is the current evidence on its functional and clinical efficacy? The results reveal the importance of systems capable of self-adjustment and the need for closed-loop control systems to adequately modulate assistance in individual conditions. Other advanced strategies, such as combining variable and constant frequency pulses, could also play an important role in reducing fatigue and obtaining better therapeutic results. The field not only would benefit from a deeper understanding of the kinematic, kinetic and neuromuscular implications and effects of more promising assistance strategies, but also there is a clear lack of long-term clinical studies addressing the therapeutic potential of these systems. This review paper provides an overview of current system design and control architectures choices with regard to their clinical effectiveness. Shortcomings and recommendations for future directions are identified.

## Background

**Fig. 1:**
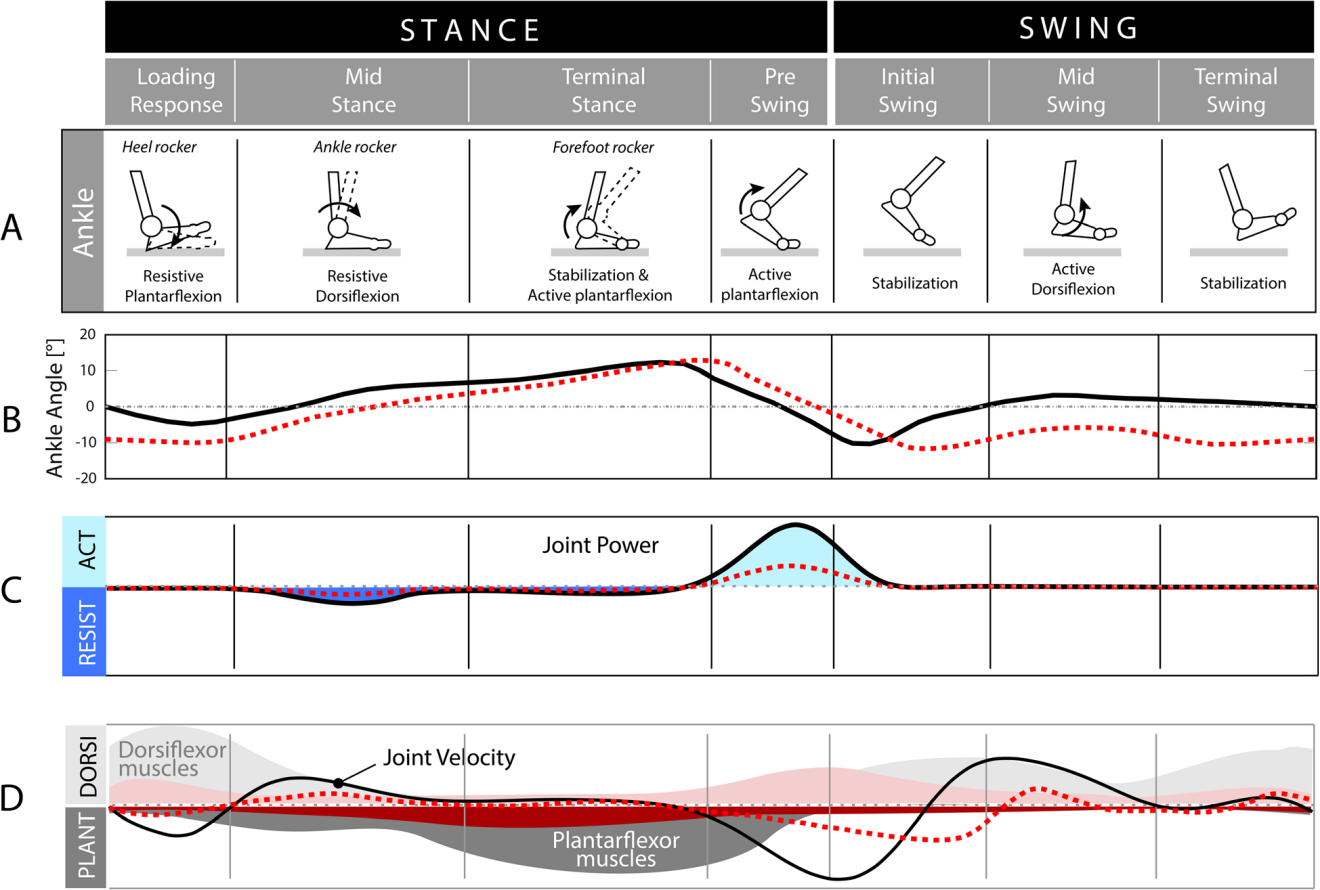
Biomechanics of the ankle in the gait cycle, musculature and nerves. The graphs represent the biomechanics of the ankle of a healthy subject (black continuous line) versus the biomechanics of a subject with foot drop (red segmented line). An example of muscle activity in foot drop (taken and adapted from [3]) is plotted in panel D (red)

Most of the neurological impairments affecting gait, such as a cerebrovascular accident (CVA) or stroke, spinal cord injuries (SCIs), multiple sclerosis (MS), cerebral palsy (CP), and brain injuries (BIs), occur at significant incidence rates globally [1, 2]. Foot drop (FD) is a common gait impairment derived from these pathologies, which consists of a paralysis or significant weakness of the ankle dorsiflexor muscles. It is characterized by the inability to achieve an adequate dorsiflexion, as shown in Fig. 1b, to obtain a sufficient distance with the ground during the swing phase of gait [4]. As a result, it can lead to inefficient gait compensations (Fig. 1c and d), increase falls, greater energy expenditure, and reduced endurance [5]. It is also characterized by an uncontrolled plantarflexion, which leads to foot slap. As a result of muscle weakness and/or spasticity, individuals with FD may also become unable to support their own weight [4]. It is therefore vital to identify appropriate strategies of intervention to overcome foot drop symptoms and improve gait [6]. Conventional treatment involves the use of an ankle-foot orthosis (AFO), which keeps the ankle joint in a neutral position [7]. However, techniques based on robotic and/or electrical stimulation assistance are being developed and represent promising alternatives.

To choose the most appropriate FD treatment, it is important to take into consideration its causes and severity of the as well as the pre- and post-operative conditions of the patient. Figure 1 depicts profiles of gait biomechanics in intact humans compared to an example of a patient with FD. An inspection of gait biomechanics is relevant to establish the joint and muscular alterations that are to be reestablished or compensated. On the other hand, it is necessary to pay attention to the central or peripheral origin of the pathology, since treatment choice may vary depending on whether the first or second motor neurons are affected [8].

Neuromuscular electrical stimulation consists of the application of an electrical current through electrodes placed above the motor point to achieve a muscle contraction. It is achieved when the stimulation applied exceeds the motor threshold. The most common techniques to compensate FD are *functional electrical stimulation* (FES), which sequentially activates paralyzed muscles through electrical stimulation to restore the functional movement and is clinically advantageous in gait restoration, or *transcutaneous electrical nerve stimulation* (TENS), which is a non-invasive technique that is usually used as analgesic treatment [9]. These two techniques have different effects on FD, but in both of them the second motor neuron must be intact and the electrical excitability in the peripheral nerves and muscle tissues must be preserved [10]. FES is usually applied to increase dorsiflexion force, with a reduction in the muscular tone and a stiffness of the gastrocnemius. TENS is effective in reducing pain and increasing presynaptic inhibition, resulting in reduced spasticity, muscle tone, and stiffness. Functionally, FES has demonstrated significant effect on the spatiotemporal parameters of the gait, whereas TENS has not reported positive results yet [9, 11].

The first neuroprosthesis based on FES was developed in 1961 by Liberson et al. [12]. It was controlled by a foot switch that activated a peroneal nerve stimulation during the swing phase [13]. Since then, numerous systems have been developed to stimulate the tibialis anterior (TA) or common peroneal nerve (CPN) during the swing phase to ensure an adequate dorsiflexion, allowing the necessary foot clearance [14]. Subsequently, many other systems have been designed and developed that share a common architecture integrating a wearable sensor set and stimulation hardware embedding a control algorithm [4]. Enabling daily and unsupervised use of this type of systems is crucial for its success. These implies that systems need to be easy to place, adjust and use. In addition, they must be able to properly assist and adapt the electrical stimulation according to the muscle response that is time-varying, non-linear and coupled [15]. Ideally, the use of a non-invasive neuroprosthesis must target both compensatory (gait facilitation) and rehabilitative effects. In other words, on the one hand, the use of a neuroprosthesis must improve the biomechanics of gait and facilitate this activity. On the other hand, a long-term rehabilitation must be intended to promote recovery towards a more physiologically autonomous gait without neuroprosthetic assistance.

As far as the compensatory effect is concerned, an FES-based neuroprosthesis for FD correction must be able to achieve a sufficient distance between the floor and foot during the swing phase through a correct dorsiflexion of the ankle, as well as reduce the foot slap produced during the load response phase owing to uncontrolled plantar flexion [4]. However, it is important to note that it FES assistance of the dorsiflexors alone also can decrease the knee flexion and ankle plantar flexion at the toe-off. As a result, the propulsive force generated during a pre-swing has also been shown to decrease [16]. A possible solution to this may be plantar flexion assistance with FES during the pre-swing, which improves the knee flexion during the swing and enhances the propulsive force during the push-off [17]. Moreover, the stimulation time can be prolonged after contact with the ground to avoid a sudden plantar flexion [18].

Although non-invasive neuroprosthetic technologies for human walking continue to advance and their functional benefits for neurologically injured subjects have been demonstrated, technological barriers remain regarding the wider and sustainable adoption of such systems by patients. Although several reviews have been released to date (e.g. [4]), none of them provided a thorough analysis of the rehabilitation potential of the existing solutions. This perspective is in our opinion necessary to analyze the viability of the different advances in the face of real use in clinical and daily life settings.

This review addresses the most relevant FES assistance systems for FD of the last two decades, with special emphasis on the control architecture and its clinical effectiveness toward the most common pathologies affected.

## Methods

We carried out a search on the following databases: PubMed, PEDro, SCOPUS, Academic Google, MEDLINE, EMBASE, ResearchGate, WoS, and SciELO were consulted. The search keywords were “FES system,” “drop foot,” “foot drop,” “ankle,” “gait,” “efficiency,” “neuroprosthesis,” and “clinical results.” The criteria for inclusion were:

- Studies between 2000 and 2018.
- Studies presenting an FES system for FD with details regarding the architecture and/or clinical results.
- Studies presented in a journal or conference.
- Thesis or catalogue.

The exclusion criteria were as follows:

- Hybrid orthoses using robotic actuation in combination with electrical stimulation.

**Fig. 2:**
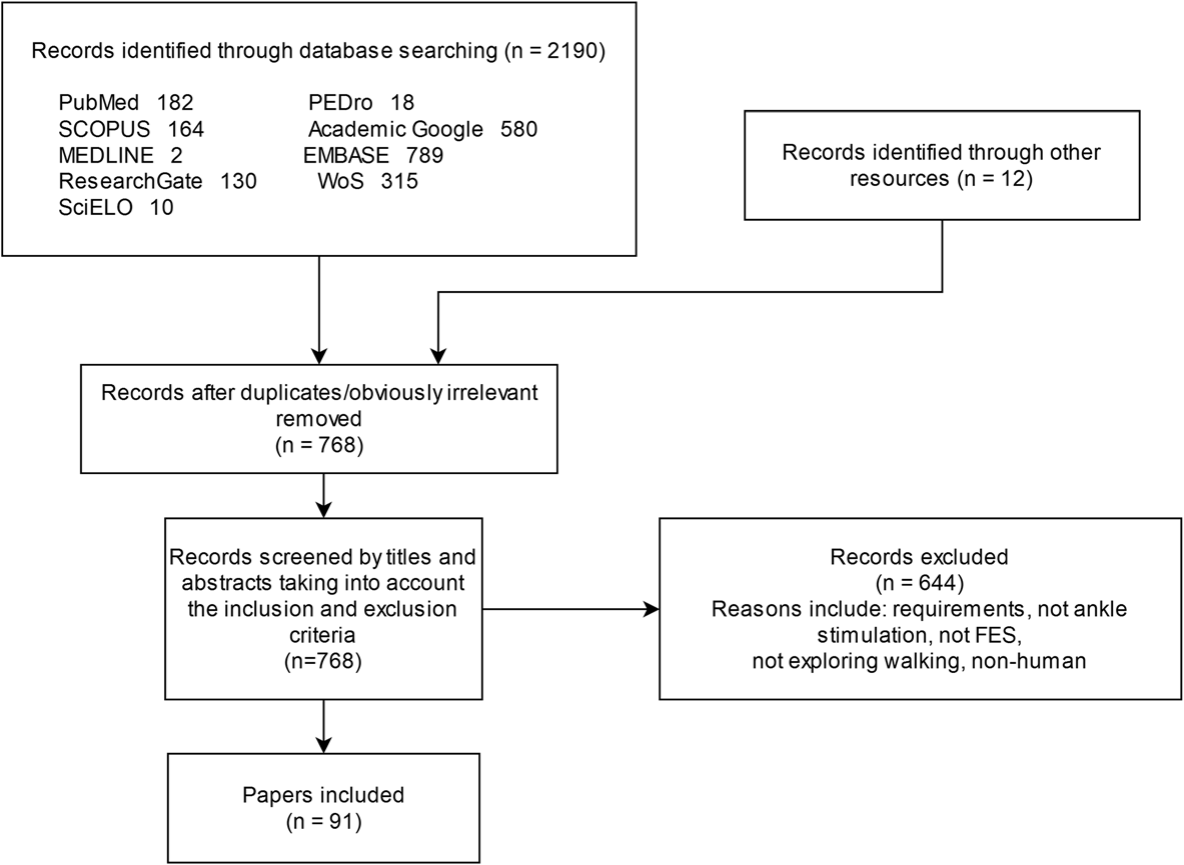
Flow diagram for procedure followed

As shown in Fig. 2, this search strategy revealed 91 articles. In addition, the final list of selected articles included others reporting on functional electrical stimulation and foot drop pathology to lay the foundation for these concepts.

## Basis for design of foot drop neuroprostheses

**Fig. 3:**
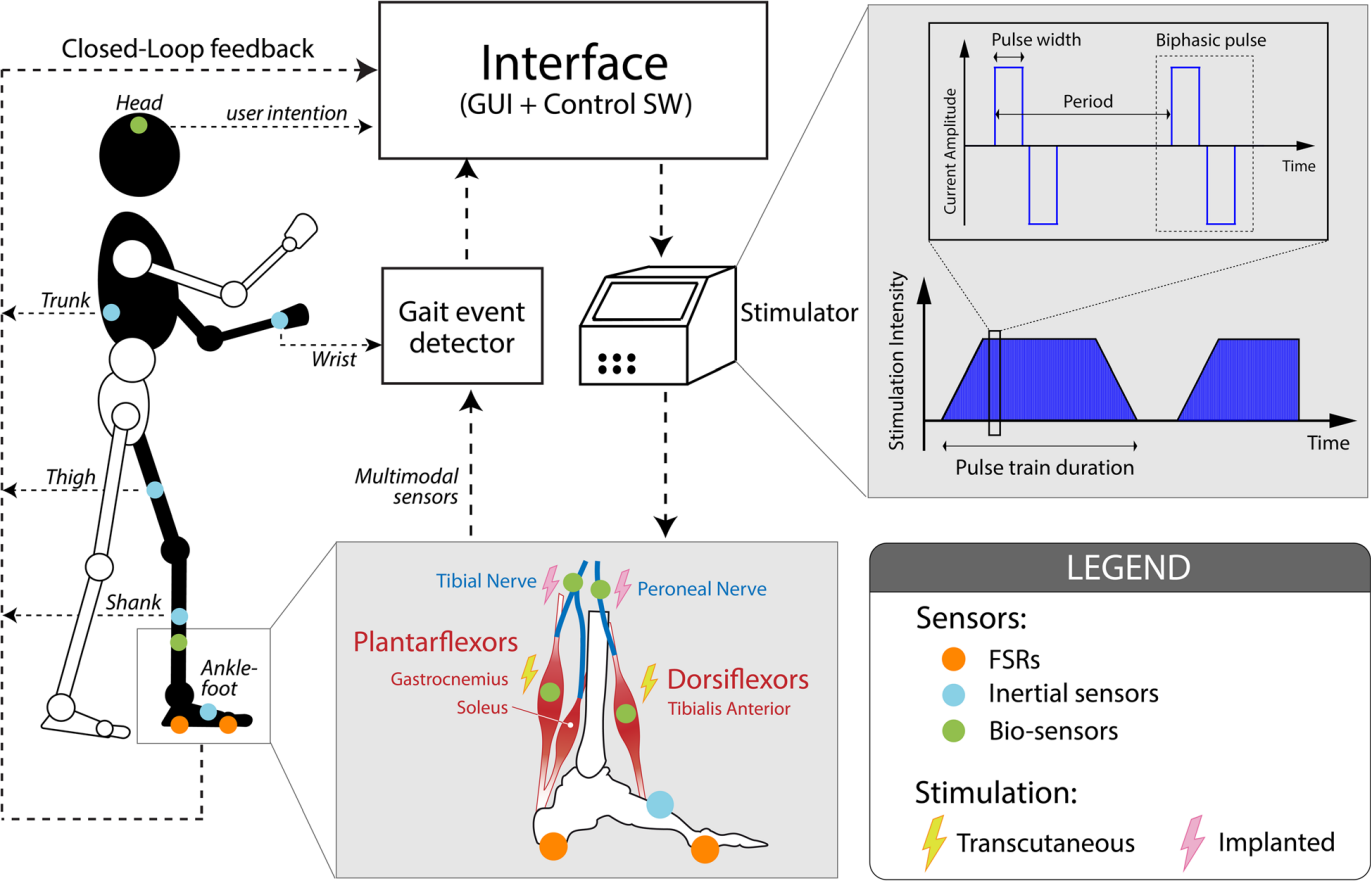
Architecture of a FD neuroprosthesis. This figure shows the sensors that have been used in the last two decades, as well as their location on the body. It also details the stimulation parameters and where assistance is normally applied

This section provides an overview of the basic fundamentals required for the design and use of a neuroprosthesis whose architecture is reflected in Fig. 3. Specifically, the methods found for (a) establishing an optimal electric current technique that allows a minimally aggressive effective assistance to be applied to the foot drop (section 3.1), (b) detecting gait events and using that information to control the timing of the applied stimulation (section 3.2), and (c) controlling the supply of electric stimulation during gait to adjust the applied electric stimulation to achieve the desired effect in an effective and optimal manner (section 3.3) are detailed.

### FES technique

FES uses electrical pulse trains on the muscle or peripheral nervous system to trigger a controlled tetanic muscle contraction [9, 10, 19–21]. The shape of the individual pulses that make up the electric pulse trains has an effect on the muscle response. They are usually rectangular as they are the most efficient in generating muscle contractions, aiming at a reduction of the habituation effect. Pulse trains must provide an equal distribution of charges within the tissue to avoid an electrochemical imbalance, which produces its damage. This is generally achieved by applying one pulse during the positive phase and another during the negative phase, symmetrical or not [4]. In this way, such pulses can be distinguished as monophasic (positive phase only) and biphasic (both positive and negative phases) [10]. Monophasic pulses create charge imbalances because of a unidirectional current flow. By contrast, biphasic pulses allow the application and removal of electrical charges to and from the tissue, and thus the majority of neuroprostheses use biphasic pulses [22].

Apart from the shape, pulse trains are described through the following five parameters, all them having an influence on the stimulation effects: the amplitude or intensity of the pulses, the frequency or repetition rate of the pulses, the duration of a single pulse, the duration of a pulse train, and the stimulation pattern or disposition of the pulses within a stimulation train [10, 19]. The first two parameters are the primary parameters that are modulated to control the movements and are related to the intensity of the contraction and fatigue [19, 23].. By modulating the amplitude of the pulses that compose a train of pulses, different wave profiles can be obtained. This is usually trapezoidal with a ramp up and down, the adjustment of which influences the strength and comfort of the stimulation and avoids a sudden response [4]. In terms of frequency, the most commonly used are between 20 and 50 Hz [18, 23]. The width of the pulses has a direct effect on the intensity of the contraction and therefore in the fatigue [24–26]. Moreover, in relation to stimulation pattern, pulse trains can be classified into two types according to the inter-pulse intervals: constant frequency trains (CFTs) and variable frequency trains (VFTs). CFTs is often used and consists of stimulation pulses separated by constant inter-pulse intervals. In VFTs, the inter-pulse intervals are not constant [5, 27].

Electrical muscle and nerve stimulation using this technique can be achieved with surface (transcutaneous) or internally implanted electrodes (Fig. 3). A superficial stimulation allows for application flexibility, whereas implanted electrodes entail higher risks for the patient in favor of better selectivity [19]. Depending on the location of the electrodes, the electrical stimulation can be applied to both muscles and nerves. The main difference is that the electrodes placed on muscles produce the activation of a single muscle, whereas the electrodes located in a nerve can activate multiple muscles simultaneously [28].

Electrostimulators share that they must be portable and lightweight and allow each muscle group to be activated according to the time, duration and intensity of a pre-programmed individual stimulation. However, differ mainly on number of channels [4, 27]. Multichannel stimulation is advantageous over a single-channel stimulation in different respects [27]. In multichannel systems (Fig. 3), it is possible to have a single anode and several independent cathodes, or to have anodes and cathodes that are galvanically isolated [10]. Moreover, multichannel systems allows to address fatigue in three stimulation modalities: synchronous or conventional, sequential and asynchronous. In conventional systems, the pulses of all channels are sent at the same time. In sequential systems, the trains of pulses are sent sequentially channel after channel, offering the opportunity to stimulate each muscle attached to each channel sequentially. In asynchronous stimulation, different channels are activated in random order, including the possibility of activating a group of channels synchronously while the rest of them in a random order or overlapping activation periods between channels. In this way, with sequential and asynchronous stimulation a low frequency per channel is achieved, maintaining a high frequency of compound stimulation [29].

### Gait event detection

Gait events must be accurately detected to allow the correct application of FES to control FD. Liberson’s design proposed the use of a heel-switch that triggers the FES assistance when a heel-off occurs. This solution opened a number of challenges, including the need to adequately adapt the stimulation according to the muscle response [2]. Since then, diverse types of sensors have been used for the correct gait event detection. A typical and simple solution is the use of force sensitive resistors (FSRs), as shown in Fig. 3. The placement under the heel and forefoot of both feet allows a detection of the heel strike, heel-off, and toe-off in real time [30]. This allows adequately detecting gait phases as a stance or swing, as well as the double support of the loading response and push off. However, this solution has certain disadvantages because it is not possible to detect the swing sub-phases and the accuracy and reliability depend on the location of the FSR sensors [2]. Alternatively, hand switches can be used to apply assistance by voluntary control of the operator [31], but this solution has only been explored in few cases in the literature.

A widespread solution is the use of inertial sensors (Fig. 3), which include accelerometers, gyroscopes, and magnetometers. These solutions allow increasing gait segmentation in further states, such as swing sub-phases. In addition, they can be used to create closed-loop control algorithms calculating the kinematic parameters. The placement and number of these sensors depend on the control algorithm, although they are usually positioned in the thigh, shank, or foot. An advantage over sensors like FSRs is that their correct operation will not vary depending on the type of footwear used or when the surface on which you walk varies. Another difference with FSRs is that there is no need to use external wiring from the sensor to the controller. The combination of all 3 types of inertial sensors has resulted in inertial measurement units (IMUs) [32, 33].

The combined use of FSRs and IMUs brings redundancy that allows in general a more robust detection of gait events, as well as the design of more reliable closed-loop FES control [32].

Another alternative is the use of electromyography (EMG), although such information is highly complex during acquisition and post-processing. This solution brings also the potential to infer parameters that are related to muscle fatigue [34]. However, an evaluation of the muscular activity of an EMG is complicated in the presence of an FES because the elimination of artifacts has yet to be perfected [35].

### FES control

Control strategies are applied to mimic or assist the functions performed by the central nervous system and activate the musculature so that natural movements can be performed. They can be classified into two modalities: open-loop or closed-loop. Both strategies use gait events data to switch between states and assist appropriately. However, open-loop stimulation strategies are a simple approach that focuses on controlling the moment of stimulation. The systems that apply this type of strategies require continuous attention because they do not adapt the stimulation applied according to muscle response [36]. This has led to the development of closed-loop strategies that are more stable and robust and are able to control the position or force generated by modulating the different parameters that characterize the stimulation. The aim of these strategies is to correct model errors, internal (e.g. muscle fatigue) and external (e.g. obstacles) disturbances [4, 32] through feedback information.

All of this has led to a large part of research in recent years focusing on the development of this type of strategy. In fact, several authors have shown that the application of closed-loop control techniques to control the ankle movement improves the regulation of muscle activation, and such techniques are able to cope with muscle fatigue and external disturbances [4, 18]. However, despite the technological development and progress achieved in closed control, for the time being, they remain under investigation and the commercial FES lower limb support systems are based on open-loop control [32].

The first FES open-loop system was proposed by Liberson et al. [12]. Open-loop control approaches are a straight-forward way to deliver FES assistance that, although achievable, has restricted flexibility, which in turn results in a non-optimal performance (with respect to a reference healthy gait pattern) and are not prepared to minimize nor control the induction of muscle fatigue [31]. By contrast, more ambitious FES applications require the ability to modulate the pulse-to-pulse electrical stimulation while walking to intelligently compensate for fatigue, spasticity, learning effects, and external (e.g., environmental) disturbances. Thus, a range of closed-loop controllers have been postulated as promising algorithmic solutions for efficient gait neuroprosthetic systems, relying on multiple types of sensor modalities, including kinematic, muscular (electromyographic (EMG) signals) and nervous (electro-neurographic (ENG) signals) activities. To summarize, the following types of control strategies have been applied for control: finite state control (FSC), iterative learning control (ILC) [37], proportional-integral-derivative (PID) control [38], artificial neural networks (ANNs) [39], fuzzy networks [40], and iterative error-based learning [41].

## FES systems for foot drop correction

**Fig. 4:**
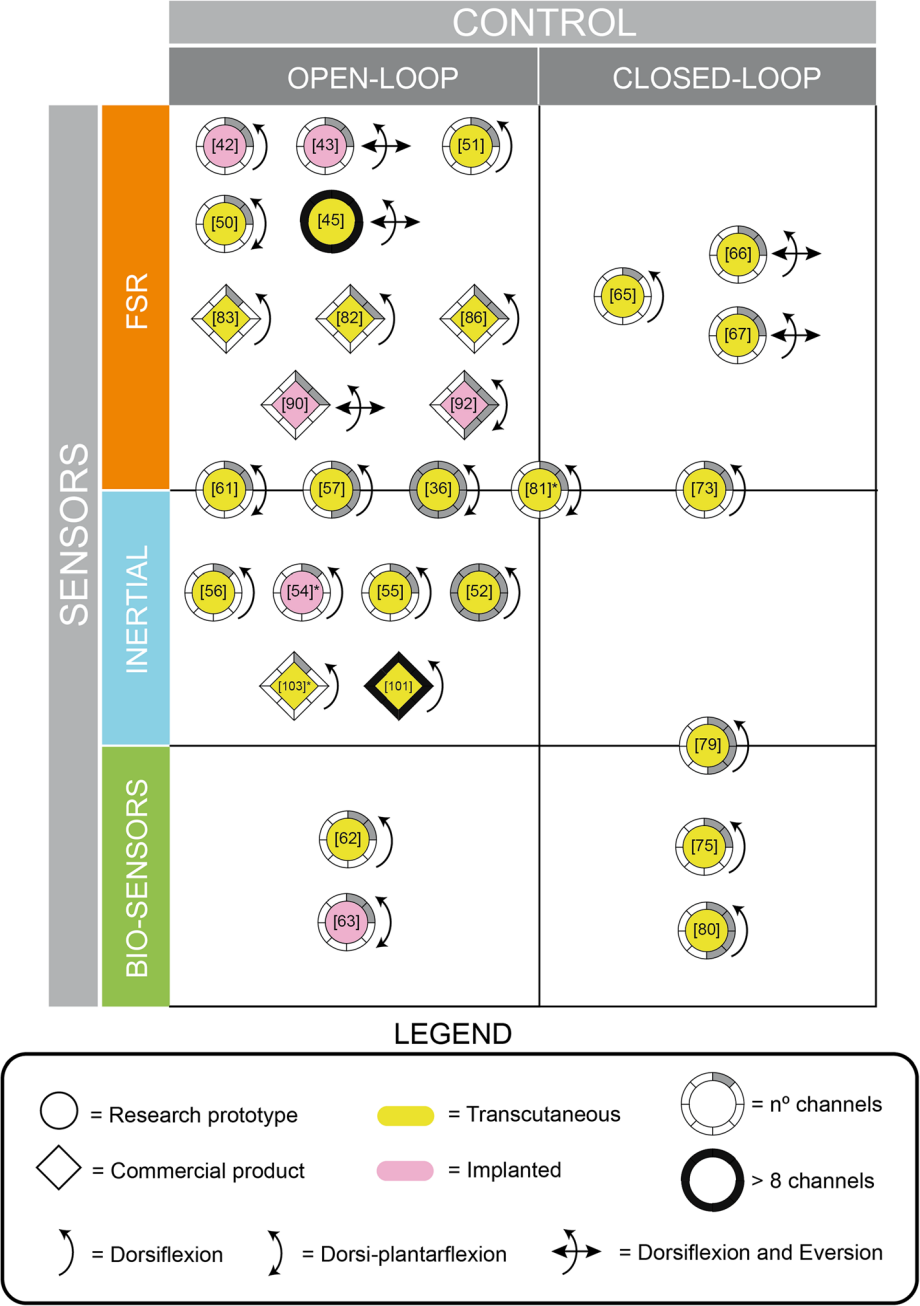
Classification of FD neuroprostheses included in this review. Type of sensors, control approach, number of available stimulation channels, type of electrodes and application type are summarized with references in brackets inside the descriptive knobs

This section reports a brief description of all the systems included in our review, organized according to a taxonomy that considers the control type (open-loop vs. closed-loop) as primary category, and gait detection method and readiness level (research prototype vs. commercial device) as secondary categories. The works are ordered chronologically in each subsection. A schematic summary is reported in Table 1. In addition, a graphical scheme summarizing this classification is provided in Fig. 4.

**Table 1:** Foot drop FES systems

Devices	Sensors	Transcutaneous/Implanted	# of Channels	Assistance	Muscles/nerves
**Open-loop systems: Research prototypes**					
Haugland et al. [42]	FSRs	Implanted	2	Dorsiflexion	Peroneal nerve
Kottink et al. [43, 44]	FSRs	Implanted	2	Dorsiflexion, eversion	Peroneal nerve
ShefStim [45–49]	FSRs	Transcutaneous	64	Dorsiflexion, eversion	Multiple muscles
Perumal et al. [50]	FSRs	Transcutaneous	2	Dorsiflexion and plantarflexion	Flexor-extensor muscles
Sabut et al. [51]	FSRs	Transcutaneous	2	Dorsiflexion	Peroneal and anterior tibial nerve
ExoStim [52, 53]	Inertial	Transcutaneous	8	Dorsiflexion	Unspecified
BIONic WalkAide [54]	Tilt sensor	Implanted	1	Dorsiflexion	Tibialis anterior and peroneal nerve
Ismail et al. [55]	Inertial	Transcutaneous	2	Dorsiflexion	Peroneal nerve
Watanabe et al. [56]	Inertial	Transcutaneous	1	Dorsiflexion	Tibialis anterior and peroneal nerve
Compex Motion [57–60]	FSR + Inertials	Transcutaneous	4	Dorsiflexion	Tibialis anterior and peroneal nerve
Gait MyoElectric [61]	FSRs + Inertials	Transcutaneous	2	Dorsiflexion and plantarflexion	Flexor-extensor muscles
Runbot III and II [36]	FSRs + Inertials	Transcutaneous	8	Dorsiflexion and plantarflexion	Tibialis anterior, lateral gastrocnemius, biceps femoris and rectus femoris
Do et al. [62]	EEG	Transcutaneous	2	Dorsiflexion	Peroneal nerve
NeuroStep [63, 64]	Neural clamps of electrodes	Implanted	2	Dorsiflexion and plantarflexion	Tibialis anterior and peroneal nerve
**Closed-loop systems: Research prototypes**					
Chen et al. [65]	FSRs	Transcutaneous	1	Dorsiflexion	Tibialis anterior muscle
DeltaStim [66]	FSRs	Transcutaneous	2	Dorsiflexion and eversion	Peroneal and anterior tibial nerves
APeroStim [67–72]	FSRs	Transcutaneous	2	Dorsiflexion, eversion and inversion	Tibialis muscle and fibularis longus
Duo-STIM [73, 74]	FSRs + Inertials	Transcutaneous	2	Dorsiflexion	Unspecified
Li et al. [75–78]	EMG	Transcutaneous	2	Dorsiflexion	Tibialis or medial gastrocnemius muscles
RehaMove Pro [79]	Inertial + EMG	Transcutaneous	4	Dorsiflexion	Unspecified
Nahrstaedt et al. [80]	Electrodes to measure bioimpedance	Transcutaneous	4	Dorsiflexion	Dorsiflexors muscles
**Combined systems**					
O’Keeffe et al. [13]	FSRs, Inertials, EMG and electrogoniometers	Unspecified	2	Dorsiflexion	Unspecified
Melo et al. [81]	FSRs + Inertials	Transcutaneous	2^a^	Dorsiflexion and plantarflexion	Flexor-extensor muscles
**Open-loop systems: Commercial prototypes**					
MyGait [82]	FSRs	Transcutaneous	2	Dorsiflexion	Peroneal nerve
Odstock [83–85]	FSRs	Transcutaneous	1	Dorsiflexion	Unspecified
NESS L300 [86–89]	FSRs	Transcutaneous	2	Dorsiflexion	Tibialis anterior and peroneal nerve
STIMuSTEP [90, 91]	FSRs	Implanted	2	Dorsiflexion, eversion	Peroneal nerve
ActiGait [73, 92–100]	FSRs	Implanted	4	Dorsiflexion and plantarflexion	Peroneal nerve, tibial and peroneal muscles
FESIA WALK [101, 102]	IMUs	Transcutaneous	Multi-pad	Dorsiflexion	Tibialis anterior and peroneal nerve
WalkAide [103–112]	Tilt sensor	Transcutaneous	1	Dorsiflexion	Tibialis anterior and peroneal nerve

^a^ Note: the system is reported to be modular and can scale up the number of sensors

### Open-loop systems: research prototypes

#### Stimulation based on foot switches

Many of the systems based on Liberson et al.’s open-loop control architecture used heel switches to detect gait events. One example is the system developed by Haugland et al. (2000), who proposed an implanted two-channel stimulator and an external control unit based on a microcontroller, powered by rechargeable NiMH batteries. This system stimulates the peroneal nerve through clamps of multipolar electrodes implanted in the knee area around the same nerve. The stimulation frequency, pulse width, and ramp characteristics are adjustable. The system, very quick to be worn, was tested on three post-stroke hemiplegic individuals in home-based environment, showing good improvements in dorsiflexion. Unquantified gait velocity increase was observed during FES-assisted walking, but no therapeutic effects were reported [42].

In 2007, Kottink et al. proposed an implantable two-channel peroneal nerve stimulation system with bipolar intraneural electrodes located below the epineurium of the superficial peroneal nerve (eversion) and below the epineurium of the deep peroneal nerve (dorsiflexion). It was tested on 29 stroke patients, showing a significant 23% improvement in walking speed. No therapeutic effect was found, reflected by no changes in walking speed when the stimulator was switched off [43, 44].

The ShefStim system, developed by Reeves et al., is a 64-channel transcutaneous stimulation system composed of a stimulator and a PC. This system can stimulate multiple muscle groups and can be successfully used in an unsupervised manner thanks to a three-level automated setup procedure. The first level allows to identify the motor threshold from a short low-amplitude burst that progressively increases. The second level localizes the sensitive regions of interest by means of non-tetanic contractions. During the third phase, a tetanic test of the candidate regions is carried out through a score based on a cost function. Finally, the candidate region and the intensity of the electrical stimulation are fine-tuned with the help of participant subjective opinion. ShefStim has been clinically studied on 32 patients post-stroke, 34 patients with MS, and one patient with brain trauma for lower limb rehabilitation. Results show increased walking speed (from 10 to 23, or 36%), reduction in walking effort (Borg scale score ~ 9), improved dorsiflexion and reduced inversion at the initial contact. In addition, this work was the first in demonstrating the feasibility of using an FES system outside of the laboratory environment without technical support [45–49].

In 2009, Perumal et al. presented a transcutaneous system that uses foot switches located under the heel and toe are used to detect gait events. The stimulation is composed of variable-frequency trains (VFTs) applied in the flexor-extensor muscles of the ankle to produce dorsiflexion in the rolling phase and plantar flexion in the final phase of double support. Dorsiflexors were stimulated using a VFT composed of an initial three pulses of 200 Hz followed by a 30-Hz constant frequency train (CFT). In the case of plantar flexors, two logics designed to increase the plantar–flexor force during a push-off were compared. Logic 1 was composed of a 20-Hz CFT during the early stance phase to decelerate the tibia, and a 30-Hz VFT during push-off. Logic 2 was composed of a 30-Hz VFT applied in the terminal stance of the paretic leg followed by a 20-Hz CFT until a toe-off of the paretic leg was detected. Four post-stroke individuals with hemiparesis participated in the study. Results showed improved dorsiflexion during swing, and indirect effects on posterior ground reaction force and knee flexion during swing as a results of plantar flexor stimulation [50].

A year later, Sabut et al. (2010) developed and evaluated a two-channel transcutaneous stimulation system, applied to the peroneal and tibial anterior nerve. The system was tested on a subject with CVA during a 15–30-min walking session per day over a period of 4 weeks. Results showed that walking speed increased by 27.27%. The root mean square value of EMG of the TA also increased by 50 and 37.5% in the same way. Results also showed improved ankle range of motion (ROM) [51].

#### Stimulation based on inertial sensors

In 2004, Simcox et al. presented system with a portable eight-channel surface stimulator, called ExoStim. This system used orientation sensors and control and adjusts all stimulation parameters in real time. No experimental results have been found, although as detailed, they would be validated in future studies [52, 53].

In the same year, Weber et al. proposed an implanted minimally invasive stimulation system called BIONic WalkAide and based on the combination of WalkAide system and BION micro-stimulators (BIOnic Neurons). This system uses tilt sensors for gait events detection. These sensors are a combination of accelerometers and inclinometers to measure speed and position. The muscular activation for dorsiflexion is carried out through the peroneal nerve. Few measurements have been made with the system, but it seems to improve the walking speed (iSCI patient: 9,4 m/min and implanted stimulation: 17,8 m/min), obtaining results similar to superficial stimulation (surface stimulation: 19,6 m/min) [54].

Ismail et al. (2015) presented a transcutaneous stimulation arm-control system consisting of two main units: a wrist-arm balancing unit, i.e. an accelerometer, which detects the motion of the arm swing and predict the gait step intention, and a calf unit with a standard electronic muscle stimulation system that applies the stimulation to the peroneal nerve. This system was tested on six healthy subjects and one patient with CVA. Pilot test results showed an improved walking speed (25%), increased cadence (7.96 step/min), increased step length (7 cm), and a good foot clearance during a swing phase [55]. No other studies have been found that highlight more results or therapeutic effects.

Watanabe et al. (2016) developed a prototype of a portable single-channel transcutaneous stimulation system composed of a tablet interface, two inertial sensors located on the paretic foot (optionally on the other limb) that send information regarding gait events through Bluetooth to the PC and the stimulator. The stimulation was applied to the TA and CPN simultaneously. No results have been reported [56].

#### Stimulation based on FSR and inertial sensors

One of the most widespread strategy to detect gait events and control FES assistance is combining FSR with inertial sensors. Popovic et al. (2001) presented a portable and programmable four-channel transcutaneous stimulator system called Compex Motion, which used three FSRs and a gyroscope to detect the gait events. An open-loop stimulation is applied to the dorsiflexor muscles (TA and CPN) during the swing phase. The device has been applied to patients with CVA or SCI but no results have been reported. The stimulator has not been made commercially available because Compex SA decided not to enter this market segment [57–60].

Embrey et al. (2010) presented the Gait MyoElectric stimulator, which is a two-channel transcutaneous stimulation system that applies stimulation to the flexo-extensor muscles and uses FSRs placed in the heel and forefoot of the non-paretic leg and an accelerometer to gait events detection. It was tested on 28 CVA patients and the results showed a significant increase of 32.7% in the dorsiflexion strength in the paretic leg after 3 months of training. Moreover, results suggested that the combination of flexo-extensor muscle stimulation with 1 h of walking each day translates into better walking without FES-assistance in patients with a chronic hemiplegia [61].

Meng et al. (2017) presented Runbot II and III, two versions of a transcutaneous FES system which use IMUs and FSRs placed under the heel and the first metatarsal for gait event detection. The stimulation was applied to the tibialis anterior, lateral gastrocnemius, biceps femoris, and rectus femoris. Results showed an increment in plantarflexion during pre-swing and dorsiflexion in swing. A decrease in knee extension during a stance and an increase in the knee flexion during a swing and extension during heel contact were also observed [36].

#### Stimulation based on EEG

Do et al. (2011) proposed a non-invasive system that uses a brain computer interface (BCI) to control FES stimulation. EEG patterns are detected in real time and the system allows a personalized model for dorsiflexion prediction to be developed through a training procedure with a precision reaching as high as 97.6%. This system employs a low-consumption constant-current neuromuscular two-channel stimulator. The stimulation was applied to the peroneal nerve. The system was validated in five healthy subjects, but it was thought that patients could use it based on an ipsi-lateral paradigm, where the attempted movement of the affected extremity acts as a control [62].

#### Stimulation based on ENG activity

The NeuroStep system is a fully implanted stimulation system composed of a control unit and two neural clamps of electrodes (Neurocuffs) implanted around the tibial and peroneus nerves above the knee. Stimulation is applied in a closed-loop controlled using real-time ENG signals of the tibial nerve. This system was originally designed by Hoffer et al. [63] and assisted dorsiflexion during the swing phase via peroneal nerve stimulation. Later, Atsma et al. [64] upgraded that technology to include assistance of plantarflexion via tibial nerve stimulation. This allows determining when the hemiparetic foot is in contact with the ground. It specifically detects a heel contact and toe lift. This information is also used to detect when the subject is standing up to activate the assistance system and control the foot trajectory during a swing motion. The system was used by six subjects with FD. The results of Atsma et al.’s group with the second version of the system revealed that a correct ankle dorsiflexion and plantar flexion with gastrocnemius has the potential to produce a knee flexion moment with a destabilizing effect in patients. However, using this FES system, the plantar flexion was sufficiently strong to cause a heel lift through a tibial nerve stimulation [63, 64].

### Closed-loop systems: research prototypes

#### Stimulation based on FSR sensors

Chen et al. (2013) developed a real-time transcutaneous self-adaptive system. This system uses an FSR sensor placed under the heel to estimate the step frequency and predict the swing phase from previous steps. They use the TA EMG recorded from 10 healthy individuals walking over ground to build up a database of swing-stance profiles stimulation envelopes. The system select a predefined pattern according to the swing phase duration. Then, the stimulation is applied over the entire gait cycle using pulse width modulation (PWM), where the width of each pulse is proportional to the corresponding intensity of the selected envelope. This system was applied to eleven healthy subjects and one post-stroke inpatient to evaluate the precision of the prediction model, but no experimental results of gait performance have been found. The stimulation was applied to the TA. Results showed that a higher velocity makes it more difficult to predict the step frequency [65].

#### Stimulation based on inertial sensors

Valtin et al. (2014) presented a transcutaneous stimulation system composed of the DeltaStim stimulation system (HASOMED GmbH, Germany), two inertial sensors used in the foot and leg for gait event detection and a PC that allows configuring and controlling two virtual electrodes in real-time. To configure the virtual electrodes, a sequence of monophasic stimulation pulses are applied to two channels in order to define the stimulation intensity sufficient to produce dorsiflexion without discomfort. Both channels generate dorsiflexion, although the tibialis anterior muscle channel normally produces a small inversion, whereas the peroneal nerve channel produces an eversion. The virtual electrodes are automatically determined by monitoring the stimulation effects on ankle flexo-extension and eversion-inversion using two IMUs on the shank and the foot. This system allows automatic calibration to be performed with a few steps of the patient. Subsequently, the stimulation will be adjusted and controlled in a closed-loop by an ILC, which allows achieving natural physiological movements [66].

In 2014, Seel et al. proposed a transcutaneous stimulation system called APeroStim. One small 6D IMU attached to the midfoot is used to detect the events of the gait (swing phase, heel contact, and toe-off). Two or three transcutaneous electrodes are placed on the tibialis muscle and fibularis longus. The stimulation is controlled in a closed-loop by an ILC, which allows controlling dorsiflexion and eversion with respect to a given reference trajectory, adjusting the stimulation intensity between steps. In addition, a new method to detect gait phases through a single IMU in the foot has been developed, which adapts to the walking speed of the subject and the walking characteristics of stroke patients. Results have also revealed that the intensity profiles needed to produce a physiological foot movement are not typically trapezoidal. They vary in time as well as between patients [67–72].

#### Stimulation based on FSRs and inertial sensors

Breen et al. (2006) developed Duo-STIM, a portable two-channel transcutaneous stimulation system that provides real-time stimulus adjustment from cycle to cycle based on the stride time and high flexibility of the output waveform shape. It consists of the programmer unit and the portable stimulator unit. For gait event detection, it uses two FSR sensors, one placed under the heel and the other placed under the first metatarsus of the foot, and IMUs [73, 74].

#### Stimulation based on EMG

In 2015, Li et al. proposed a system composed of a wireless transcutaneous two-channel stimulator, a laptop computer with a MATLAB interface and an evoked electromyography (eEMG) (MP100, Biopac Systems Inc., Santa Barbara, CA, USA). A joint torque measurement device (Biodex 3, Shirley Corp., NY, USA) is used to verify and evaluate the performance of the prediction based on eEMG. The stimulation consists of trapezoidal trains applied to the tibialis anterior or medial gastrocnemius muscles. The closed-loop control system uses real-time eEMG information as feedback to predict the torque to be produced. In a muscle under stimulation, the eEMG containing the M wave is recorded. This wave provides information on the action potentials that can be applied to compensate for muscle fatigue and design a closed-loop control. The algorithm identifies the relationship eEMG-torque and eEMG-stimulus. With this information, it modulates the pulse width to follow a desired trajectory and the eEMG model is updated. The system was tested on able-bodied subjects and SCI patients, and the results verify its feasibility and efficiency [75–78].

#### Stimulation based on inertial sensors and EMG

Valtin et al. (2016) presented the RehaMove Pro, a four-channel transcutaneous stimulation system combined with a wireless IMU located on the foot to detect gait events. The interface of the PC or tablet allows remotely controlling the device and adjusting the stimulation parameters. The stimulation strategy is controlled using an ILC applied to single electrodes or electrode arrays. The algorithm is the same as that applied in the APeroStim project (section 4.2.2.), which allows controlling ankle dorsiflexion and eversion during swing. The sEMG activity was recorded using stimulation electrodes even during stimulation [79]. The voluntary part of an EMG signal can be extracted through a filtered EMG, which can be used as a trigger for electrical stimulation or as a stimulation intensity control. The objective in future studies is to use the information provided by the M waves and adapt the control strategy of FES proposed by Klauer et al. [113] to stimulate the musculature.

#### Stimulation based on bioimpedance

In 2008, Nahrstaedt et al. presented the Rehastim four-channel transcutaneous closed-loop FES system consisting of four electrodes for bioimpedance measurements, an interface, and an externally controllable stimulator. Bioimpedance provides information about the passive electrical properties of tissue and it is used to determine joint angles. The stimulation assists the dorsiflexors muscles and is controlled through the ILC technique. This control allows tracking the ankle trajectory during the swing phase and consists of applying a wave intensity profile during a gait cycle. Then, the error signal of the last gait cycle is used to update the wave intensity profile of the next cycle [80].

### Combined systems

O’Keeffe et al. (2002) developed a portable research FD stimulator with two independent programmable stimulation channels. Four analogue and four digital sensor input channels were provided with a wide variety of sensor types available (FSRs, EMG amplifiers, integrated accelerometers and electrogoniometers). A microcontroller allows implementing different control algorithms (open-loop and closed-loop), and a PC user-interface enables easy configuration. The stimulation parameters can be adjusted for each channel. This system allows to choose between monophasic and biphasic pulses, including up and down stimulation ramps and singlet, doublet or triplet waveform. Although it is a system for foot drop assistance, no results have been found from its use in patients nor from the development of control algorithms [13].

In 2015, Melo et al. proposed a flexible low-cost microcontroller-based platform for rapid prototyping of FES neuroprostheses. This platform integrates most of the sensors used in FES gait neuroprostheses (one tri-axial accelerometer, two external 9–axis IMUs, and four external FSRs), enabling both open-loop and closed-loop control strategies. No studies have been found that show results from the use of this platform [81].

### Open-loop systems: commercial prototypes

Otto Bock HealthCare Products GmbH (2013) proposed the use of MyGait, which is a transcutaneous two-channel stimulation system composed of a remote control, a heel switch placed in a special shoe, and a clamp placed in the lower part of the leg. The stimulation can be applied bilaterally on the peroneal nerve during the swing phase to control the dorsiflexion [82].

Odstock is a single-channel transcutaneous stimulation system. A heel switch located under the user’s foot in a sock is used for the detection of gait events. The stimulation can be applied unilaterally or bilaterally. The control is dedicated to a peripheral interface controller. The system was tested in approximately 21 patients with CVA and 22 patients with MS. The results using this device indicate that the magnitude of the dorsiflexion torque is enhanced by the stimulation of the common peroneal nerve with a non-repetitive series of pulses. Stimulation with this type of pulse is based on the “catch effect” of muscles which consists of increasing the muscle tension produced by a muscle when an pulse is introduced a few milliseconds after the second initial pulse. This effect occurs in nature and has been applied in the form of “stimulation doublets” at heel rise and heel strike in the study conducted. The walking speed is also faster (5% for MS) and a significant increase in the dorsiflexion and knee flexion at initial contact and during a swing was achieved [83–85].

NESS L300 is a two-channel transcutaneous stimulation system. The stimulation is applied to the peroneal and anterior tibial nerves. An FSR is used under the sole of the shoe for the detection of gait events. It was tested in 85 patients with CVA. The walking speed increases owing to the increased step length. Results with this device have revealed a better performance with a two-channel FES. That is, there is a significant improvement when comparing the peroneal and thigh FES with respect to the peroneal FES alone. In addition, it was observed that the ankle and knee stimulation can lead to a more effective application of FES assistance by improving the temporal characteristics of the gait [86–89].

STIMuSTEP is an implanted two-channel stimulation system with bipolar implanted electrodes and a heel switch to detect gait events. The stimulation is applied to the CPN using a predetermined delay after a heel lift and extends until the heel contacts the ground. It can be stimulated either unilaterally or bilaterally. Stimulation is applied to the deep branch of the CPN to produce dorsiflexion and eversion and to the superficial branch of the common peroneal nerve to produce plantarflexion and eversion. Twenty-three people with MS used this system and all achieved an effective correction of their FD. The results showed that the walking speed (24%), distance, and endurance of the user increased. The increased speed was maintained at 3 years [90, 91].

ActiGait is an implanted four-channel stimulation system composed of an external control unit and a computer interface. It has a heel switch attached to a sock to detect the gait events, which are communicated wirelessly using the control unit. An open-loop stimulation is applied to the peroneal nerve to stimulate the tibial and peroneal muscles during a swing motion. It was tested in 50 patients to observe the complications of its surgical implantation, as well as in more than 100 patients with CVA and 14 patients with MS. The results when applied to non-progressive diseases indicate increases in walking speed (47.2%), gait endurance (51.2%), quality of life (96%) and maximum paretic ankle plantarflexion, and a reduced risk of falls. It also shows a significant improvement in peak ankle plantarflexion velocity (22%) and power (17%). Moreover, the system has demonstrated good results for progressive diseases as well. For patients with MS, it showed increases in walking speed (30.8%) and gait endurance (47%) 10 weeks after use of the system [73, 92–100].

Tecnalia R&I (Spain) developed FESIA WALK, a transcutaneous stimulation system consisting of a stimulator (FESia) that communicates through the ZigBee protocol using a PC, tablet, or smartphone where the wireless interface is located. An IMU attached to the paretic foot is used to detect gait events. The stimulation is applied using a multi-pad of electrodes placed on the skin around the patient’s knee to stimulate the common peroneal and tibial nerves. This multi-pad electrodes has 16 cathodes and 8 anodes. It was tested in 16 patients with CVA. The results indicate that the use of the FESIA WALK system combined with conventional treatment for a 4-week period may improve the gait speed [101, 102].

WalkAide is a single-channel transcutaneous stimulation system composed of a computer using WalkAide software and a stimulator. For the detection of gait events, inclination sensors (tilt sensors) are used. Stimulation is applied during the swing phase in the peroneal and anterior tibial nerves. This device was tested in 11 able-bodied individuals and 100 FD patients. The results showed an increase in walking speed over time, an increase in the range of ankle movement, a decrease in spasticity, and an increase in stability and muscle strength. The therapeutic effect after 11 months was only observed for non-progressive pathologies (e.g., CVA, SCI, head injury, and cerebral palsy) in contrast with progressive pathologies such as MS. These results indicate an important difference between these types of disorders. They reveal that the therapeutic effect continues to increase up to at least a year (by approximately 18, 28, and 38% at 3, 11, and 11 months, respectively). However, in other disorders such as MS, the therapeutic effects in approximately 3 months are (9.1% increase) and have a tendency to then decrease owing to the progression of the disease (7.9% at 11 months). Moreover, it was observed that the intermittent and short-term use of FES can be a potentially efficient, economical, and effective treatment strategy [103–112].

## Discussion

During the last decades, many different FES assistance systems have been developed for the treatment of FD, although the first concept proposed in 1961 by Liberson et al. remains extremely popular. All these systems have a typical common architecture composed of a stimulation unit, an integrated sensor network and a control algorithm. Relevant technical advances have been achieved in each of these technical components toward more robust and efficient tools. In terms of stimulation units, efforts have pointed toward portability, with lightweight devices where control can be achieved wirelessly on a screen, tablet, or smartphone. Advances have led to the development of multichannel systems that allow controlling a greater number of muscle groups, improving their applicability to a wider range of pathologies. The development of multichannel systems (Fig. 4) has also positively contributed to the management of muscle fatigue, as in the case of asynchronous stimulation. However, it does not appear to have been used in the systems developed in recent decades and this may be because its combination with closed-loop control is complicated. These stimulation strategies can play an important role and their use will depend on the requirements of the therapy applied. For example, asynchronous stimulation would be the best indicated option for therapies in which it is necessary to perform tasks or movements for as long as possible. However, when muscle strengthening is sought, conventional stimulation may be more beneficial [29, 114].

The stimulation parameters play an important role in FD neuroprostheses. Studies have revealed that a square pulse is the most efficient, because it avoids an accommodation of the membrane. Symmetrically compensated biphasic pulses are generally recommended because they allow electrochemical imbalances that produce tissue damage to be avoided [4, 22]. As for the wave profile, a trapezoidal shape (i.e. presenting an ascending and/or descending ramp) has been proven to produce in general a more comfortable assistance and a smoother movement closer to the healthy physiological patterns [4]. In contrast to this, some studies on the APeroStim have revealed that the intensity profiles required to produce physiological foot movements are not trapezoidal, and are time-variable across patients. Therefore, time-variable intensity wave profiles can be more efficient although current systems do not incorporate strategies that use them. We believe that for the development of this type of strategies, it will be necessary to use signals that provide information on muscle activation and fatigue [72].

The disposition of individual pulses has led to CFTs and VFTs, whose combination seems to have yielded interesting results for future applications, as shown in Perumal’s research [50]. In addition, some research has shown that frequency modulation may be in strengthening muscles, although it does not appear to introduce significant advantages in relation to muscle fatigue [115, 116]. Although this technique does not seem to have been used in the systems developed in recent years, we consider particularly relevant to investigate the possibilities that the combination of VFT and CFT offers for FD systems.

The advantages and disadvantages of surface and implanted stimulation have been addressed by several studies [19]. One of the key issues of transcutaneous stimulation is the correct placement of the electrodes [117]. The current trend is to use electrode arrays combined with a self-calibration system that allows the proper selection of virtual electrodes to achieve an optimal dorsiflexion movement, avoiding an eversion or inversion of the ankle during the same process. Examples of this type of systems are the ShefStim [45–49] and DeltaStim [66]. Moreover, although stimulation by implanted electrodes allows assistance to be applied in a more specific way, the use of virtual electrodes self-calibrate strategies could become an effective non-invasive alternative. This approach is not very common, but we consider it particularly promising as it can facilitate the use of the neuroprosthesis in the daily practice without technical supervision [4, 67, 118].

Most stimulation techniques have focused on the tibialis anterior or common peroneal nerve to modulate dorsiflexion during the swing phase. In addition, in many cases the duration of the assistance is extended to avoid sudden plantar flexion after heel contact [4].. However, in recent years it has been found that it is necessary to extend the assistance to other muscles and gait phases. In the first place, the control of eversion and inversion produced in parallel with dorsiflexion has been approached by systems such as the one developed by Kottink et al. [43, 44], as well as in the DeltaStim [66], APeroStim [67–72], RehaMove Pro [79], and STIMuSTEP [90, 91] systems. Second, it has been observed that plantar flexion achieved by gastrocnemius assistance during pre-swing is an important aspect that improves knee flexion during swing and propulsive force during push off [17]. Although the introduction of these advances would bring benefits to the success of FD systems, these have not been yet adopted in most available systems that still provide exclusively assistance on dorsiflexion. In our opinion this constitutes a limitation to achieve more comprehensive and far reaching stimulation approach.

The correct detection of gait phase is a crucial aspect to consider in FD systems. Hand switches have not shown to be very useful. In contrast, the (combined) use of FSRs and IMUs is considered one of the best solutions for gait events detection, with the additional advantage of easy placement and high portability. However, these sensors do not provide physiological information needed to mimic physiological muscle coordination in an appropriate way. The indirect measurements of neuronal activities through biosensors is a promising alternative to provide this type of information [119]. For instance, EEG signals (Do et al. [62]) have been mostly used in open-loop, but their use in closed-loop is a relevant and largely unexplored direction. Other sensors, such as bioimpedance sensors (Nahrstaedt et al. [80]), eEMG (Li et al. [75–78]), ENG (NeuroStep system [63, 64]) or EMG-IMU combination (Valtin et al. [79]) also offer interesting lines of research to address some currently unsolved problems, e.g. muscle fatigue. However, these approaches require more complex strategies that allow them to be used avoiding the artifacts that FES assistance can produce [34].

With respect to control approaches, open-loop controllers have been widely adopted in both research and commercial systems due to their proven control efficiency. Despite the progresses made, problems such as muscle fatigue and nonlinear responses over time still remain unsolved. To solve these problems, technological advances in the stimulation and detection of gait events has allowed the development of closed-loop control strategies. However, such strategies remain under investigation and have yet to be commercialized. ILC appears to be the most widely used approach due to its adaptability and automatic learning. The main problem with this technique is that the duration of iterations must be constant and most biomedical engineering systems do not meet these requirements. However, Seel et al. (2011) developed a theory for systems with a variable pass length to solve the problem [120]. The use of this closed-loop control strategy in future systems is therefore promising. Most existing neuroprosthetic systems are unilateral, with just a few systems developed for bilateral control (MyGait [82], Odstock [83–85] and STIMuSTEP [90, 91]). However, with the development of multichannel stimulation units and new control strategies, the number of bilateral commercial systems is expected to increase in the coming years.

Most of the revised works in the literature have assessed the performance of neuroprosthetic systems based on kinematics. In a few recent explorations, a complementary EMG observation has been shown to increase the understanding of the effects of these systems [121]. When aiming at functional and neural recovery, we believe that both the resulting muscle activations and joint/segmental kinematics need to be considered. To date, some reviews have analyzed the effects of FES-based therapies on the improvement of functional aspects such as walking speed, reduction of effort during walking and spasticity, which translates into an improvement in the quality of life [122, 123]. Another study revealed that walking speed improvement is explained by the strengthened activation of the motor cortical areas and their residual descending connections owing to the regular use of an FD stimulator [124]. Moreover, it also been shown that neural plasticity is increased when electrical stimulation is coupled with voluntary contractions in post-stroke individuals [125].

The performance of FD systems is usually assessed on horizontal level ground surfaces. This is an important limitation, since daily lives activities frequently include walking on inclined planes, uneven terrains, stairs climbing, etc.… In addition, FD systems often use strategies that modulate current intensity or pulse width. Assistances that combine modulation of these parameters could be beneficial to patients [24, 126]. These condition should be carefully addressed in future studies, in both development and testing stages.

Most of the studies have investigated the effects of the systems during or within a short amount of time after assistance. However, the study and understanding of long-term therapeutic effects are of particular importance to determine the clinical potential of these technologies. In this regard, it is worth highlighting the study proposed by Stein et al. [90], which through a long-term observation revealed differences present in progressive and non-progressive diseases. This is particularly relevant because non-progressive diseases (e.g. CVA and SCI) can benefit from therapeutic effects, whereas progressive diseases (e.g. MS) may require continuous assistance [127–129]. Furthermore, although FES is not a technique used to replace other treatments for walking limitations, in combination with conventional rehabilitation, it may have a positive therapeutic effect on gait recovery [130–138].

## Conclusion

During the last two decades a variety of FD systems have been developed for clinical application, some of them reaching commercial exploitation. Although improvements in terms of the architecture of FES assistance and the application of strategies for obtaining optimal results have been achieved, several limitations need to be addressed for their widespread application in gait compensation, and as alternative therapeutic methods. Muscle fatigue, which results from continuous FES application, still needs to be managed in a sustainable manner. Stimulation strategies should focus on assistance as needed, as well as on closed-loop controllers that are able to dynamically cope with individual user characteristics and typical variations in spatio-temporal features of an individual gait. Moreover it seems highly relevant to include in future studies the kinetic evaluation with the assessment of the effects of FD systems on humans. We provide the reader with a summary of take-home messages from this review paper (see Table 2).

**Table 2:** Take-Home Messages

Key issues	Recommendations
The adequate wave profile for a more effective stimulation is not clear	Studies on muscle synergies in healthy people can help determine these stimulation profiles, as well as the most appropriate muscle activations based on these wave profiles to achieve a movement as close to physiologically healthy
Implanted vs transcutaneous electrodes	The development of transcutaneous stimulation systems that use electrode arrays to achieve an adequate stimulation by means of an auto setup with virtual electrodes can promote and facilitate their unsupervised use, favoring the use of these systems compared to those that apply implanted electrodes
Daily FD neuroprosthesis use is crucial	The daily use is beneficial and the design and development of portable multichannel neuroprosthesis with auto setup seeks to increase it, as well as improve the assistance provided
Musculature stimulation strategy	The muscle stimulation of FD is mainly based on the assistance of the anterior tibial or peroneal nerve during the swing phase. However, the results of different studies suggest that the assistance of the plantar flexors is also of great importance in solving this pathology
Best sensors or sensors combination	The combination of FSRs and IMUs has been the most optimal solution in terms of gait event detection. Nevertheless, advances in gait event detection through an EEG or EMG can help in many cases to perform an adequate gait phase detection and in parallel control muscle fatigue
A reduction of muscle fatigue does not have a clear solution	The combination of CFT and VFT, the selection of an appropriate stimulation wave profile and the use of closed-loop control systems may have the potential to generate physiological movements by reducing the fatigue produced
Open-loop vs Closed-loop	Open-loop systems are very popular owing to their easy implementation, but do not solve muscle fatigue, nonlinear problems, or the variable response over time. Closed-loop control systems can be a solution. Currently, the most popular method is the ILC, because this control technique is able to provide such adaptability and applies automatic learning in a simple way; however, the systems based on an EMG and ENG present a potentially useful option if the processing difficulties, which are generated with artifacts introduced through the assistance, are solved
Closed-loop control systems still do not cross the trade barrier	Although they are a very promising solution, a suitable strategy for solving problems such as fatigue, non-linear muscle response and time variable has not yet been found.
Unilateral assistance is widespread	Regardless of the affected side, the design and development of FD neuroprosthetics and strategies that allow bilateral assistance can introduce significant advances in the search for optimal therapies for the treatment of FD
Most of the studies have focused on a biomechanical analysis, specifically kinematics	With novel advances, the combined use of biomechanical and EMG analyses can be useful to improve the understanding of the movement and the effects produced by the assistance
Systems are tested in ideal scenarios that are far removed from reality	In everyday life we find inclined planes, stairs and other obstacles, not just horizontal flat surfaces to walk on.
There is a lack of evidence of long-term therapeutic results	Most of the studies have focused on the instantaneous effect of the assistance or the effect in the short-term, and it is necessary to observe the implications of the use of a long-term neuroprosthesis for the design of personalized therapies that adapt to the evolution of the pathology
Non-progressive vs progressive diseases treatment	A different approach seems to be necessary when treating FD in non-progressive and progressive diseases. In this sense, the use of implanted systems in individuals with progressive pathologies plays an important role, although the development of systems with arrays of electrodes can be very relevant and replace them.

The development of transcutaneous FD systems with automatic adjustment to provide patient autonomy outside of the clinic is a particularly promising research direction. The combination of CFT and VFT is also of special interest to facilitate walking while trying to reduce fatigue. A lack of research and development in bilateral systems in comparison to unilateral systems has been detected. It is also of special importance that the systems can adapt in robust manner to the demands of gait in everyday life, not only to account for desirable adaptations of spatio-temporal features but also to enable safe ambulation in tilted surfaces and stairs negotiation. Finally, we consider of high priority to focus on long-term studies and to adopt physiological measures that can directly or indirectly measure the neuromuscular processes, in order to better understand and quantify the mechanisms behind user’s movements.

## Data Availability

The bibliographic datasets used and analyzed during the current review are available from the corresponding author upon reasonable request.

## Abbreviations

AFO: Ankle-foot orthosis
ANN: Artificial neural network
BI: Brain injury
CFTs: Constant frequency trains
CP: Cerebral palsy
CPN: Common peroneal nerve
CVA: Stroke
EEG: Electroencephalographic or Electroencephalography
eEMG: Evoked electromyography
EMG: Electromyographic or Electromyography
ENG: Neurographic
FD: Foot drop
FES: Functional electrical stimulation
FSC: Finite state control
FSR: Force sensitive resistors
ILC: Iterative learning control
IMU: Inertial measurement unit
MS: Multiple sclerosis
PFM: Pulse frequency modulation
PID: Proportional-integral-derivative
PWM: Pulse width modulation
SCI: Spinal cord injury
TA: Tibialis anterior
VFTs: Variable frequency trains
